# Possible Mechanisms for The Effects of Calcium Deficiency on Male Infertility

**DOI:** 10.22074/ijfs.2019.5420

**Published:** 2018-10-02

**Authors:** Asghar Beigi Harchegani, Ali Irandoost, Mahdiyeh Mirnamniha, Hamid Rahmani, Eisa Tahmasbpour, Alireza Shahriary

**Affiliations:** 1 Chemical Injuries Research Center, Systems Biology and Poisonings Institute, Baqiyatallah University of Medical Sciences, Tehran, Iran; 2 Department of Medical Radiation Engineering, Central Tehran Branch, Islamic Azad, Tehran, Iran; 3 Laboratory of Regenerative Medicine and Biomedical Innovations, Pasteur Institute of Iran, Tehran, Iran

**Keywords:** Acrosome Reaction, Calcium, Capacitation, Fertility, Sperm Motility

## Abstract

Calcium (Ca) is a significant element that acts as an intracellular second messenger. It is necessary for many physi- ological processes in spermatozoa including spermatogenesis, sperm motility, capacitation, acrosome reaction and fertilization. Although influences of Ca deficiency on sperm function and male infertility have been widely studied, mechanisms for these abnormalities are not well considered. Poor sperm motility, impairment of chemotaxis, capaci- tation, acrosome reaction and steroidogenesis are the major mechanisms by which Ca deficiency induces male infertil- ity. Therefore, an optimal seminal Ca concentration is required to strengthen sperm function and all steps leading to successful fertilization. Furthermore, identification of these mechanisms provides valuable information regarding the mechanisms of Ca deficiency on male reproductive system and the way for developing a better clinical approach. In this review, we aim to discuss the proposed cellular and molecular mechanisms of Ca deficiency on male reproductive system, sperm function and male fertility. Also we will discuss the valuable information currently available for the roles of Ca in male reproduction.

## Introduction

According to World Health Organization (WHO), infertility refers to the biological inability of an individual to achieve pregnancy following at least 12 month of unprotected intercourse (1). It has been estimated that approximately 15% of couples face some form of infertility (2, 3) and among them, male factor infertility plays a role in nearly 30-50% of all infertile couples (4). Many studies have reported that different factors including: varicocele, testicular failure, testicular cancer, endocrinal disorders, hormonal disturbances, genital tract infection, ejaculatory disorders, immunological factors, prolonged exposure to heat, obesity, older age, tobacco smoking, alcohol consumption, pesticides, the effect of radiation and magnetic waves, chemotherapy, occupational exposure, electronic devices, heavy metals, reactive oxygen species (ROS), genetic and chromosomal defects, dietary and lifestyle factors and different environmental pollutants (5-11) can play a role in the etiology of male infertility.

Nutritional deficiency of some traces elements is another significant factor that affects semen quality and plays important roles in the male reproductive process. Human semen contains several trace elements such as Calcium (Ca), copper (Cu), manganese (Mn), magnesium (Mg), zinc (Zn), and selenium (Se) that are necessary for metabolic processes, normal spermatogenesis, sperm maturation, motility and capacitation, as well as sperm normal function (5, 12). Therefore reduced level of these trace elements can be considered as one of the significant factors for impaired spermatogenesis, poor semen quality and male fertility (5, 13, 14). For example, numerous studies reported decreased levels of Zn, Cu and Se in seminal plasma of infertile men compared to infertile individuals (5, 15). Furthermore, seminal Zn concentration was reported to be significantly correlated with sperm counts, motility and viability (5). Another study demonstrated declined level of zinc in seminal plasma and serum of azoospermic patients compared to normospermic men (16).

Physiological concentrations of Fe and Cu in sperm and seminal plasma have exhibited a positive correlation with a variety of antioxidant markers and a negative association with lipid peroxidation (17). Potassium and sodium are also present in high levels in human seminal plasma and have a significant role in acrosom reactions (18). Mg and Ca maintain the osmotic balance and contribute in nutrient transfer. The presence of Mg^2+^ and Ca^2+^ ions is essential for sperm capacitation, acrosome reaction and hyperactive motility of spermatozoa. It has been suggested that rise in concentrations of Ca, Mg and inorganic phosphate may be effective in treatment of accessory gland function of the male genitalia (19, 20).

Ca is one of the most extensively studied elements of mammalian semen (21). It is well-known that Ca, as an intracellular and universal second messenger, is crucial for maximum motility of sperm cells, capacitation, hyperactivation, acrosome reaction, chemotaxis and fertilization processes (22). Human spermatozoa are unable to fertilize an oocyte before their maturation through the female reproductive tract. The process of fertilization and maturation is tightly modulated by some signaling cascades and Ca, which plays a critical dynamic role in this process as an intracellular second messenger. Therefore, there may be a close relationship between Ca, and human sperm function and fertility outcome.

Understanding the mechanisms in which Ca deficiency can affect sperm function and fertility is a matter of utmost importance. Although different studies have demonstrated decreased level of Ca, in semen of infertile men, mechanisms of Ca, deficiency on male infertility are not well-considered. In the following sections we will discuss critical roles of Ca, in sperm function and consequences of Ca, deficiency in semen of the both experimental animal models and human subjects. Additionally, we will review the possible mechanisms by which Ca deficiency induces impaired spermatogenesis and male infertility.

### Relationship between Ca and male infertility

Ca serves as a regulatory factor in different biological processes such as cell proliferation, protein secretion and muscle contraction (22). Most of these biological events are modulated by an intracellular Ca receptor known as calmodulin. Upon binding to Ca , calmodulin can activate different enzymes, especially protein kinases (PK), phosphatases, and phosphodiesterases (23). Na/K-ATPase and inositol 1,4,5-tripho-sphate receptor (IP3R), as an intracellular Ca store receptor, increase intercellular Ca concentration (24).

Prostate gland is a major source of Ca in human semen (25). Since Ca concentration in the prostate, seminal vesicles and epididymis is very high, numerous studies have investigated the association between Ca and male infertility (26). Many studies have reported a relationship between seminal Ca and male infertility (20, 23). The Ca channel blockers were also reported to be associated with male infertility. For example, Prien et al. observed that semen of men with hypomotility had significantly lower Ca level than men with normal motility (27).

Similarly, Wong et al. (28) reported that seminal Ca level in patients with hypomotility of sperm was significantly lower than those in fertile subjects. In another study, it has been suggested that infertile men with and without varicocele have a significant lower Ca in their seminal plasma compared to fertile men (29). Other studies have also demonstrated a positive relationship between high Ca levels and fertility in men (30). In another research, a significant reduction was found in mean of Ca concentration in seminal plasma of normozoospermic infertile men compared with fertile men; however, it was not related to infertility classification (normo-oligo, and azoospermic) (31). Nishida et al. (32) have shown that *in vitro* exposure of human sperm to low Ca level increases fertilizing ability. Therefore, these data suggest that decreased level of seminal plasma Ca can be a reason for infertility in men. Recent studies have illustrated that there is a close relationship between vitamin D (VD) and seminal Ca concentration. Blomberg Jensen et al. (21, 33) indicated that VD deficiency can be associated with decreased level of intracellular Ca and subsequently poor sperm motility, deficiency of sperm acrosome reaction and increased risk of male infertility.

**Fig.1:**
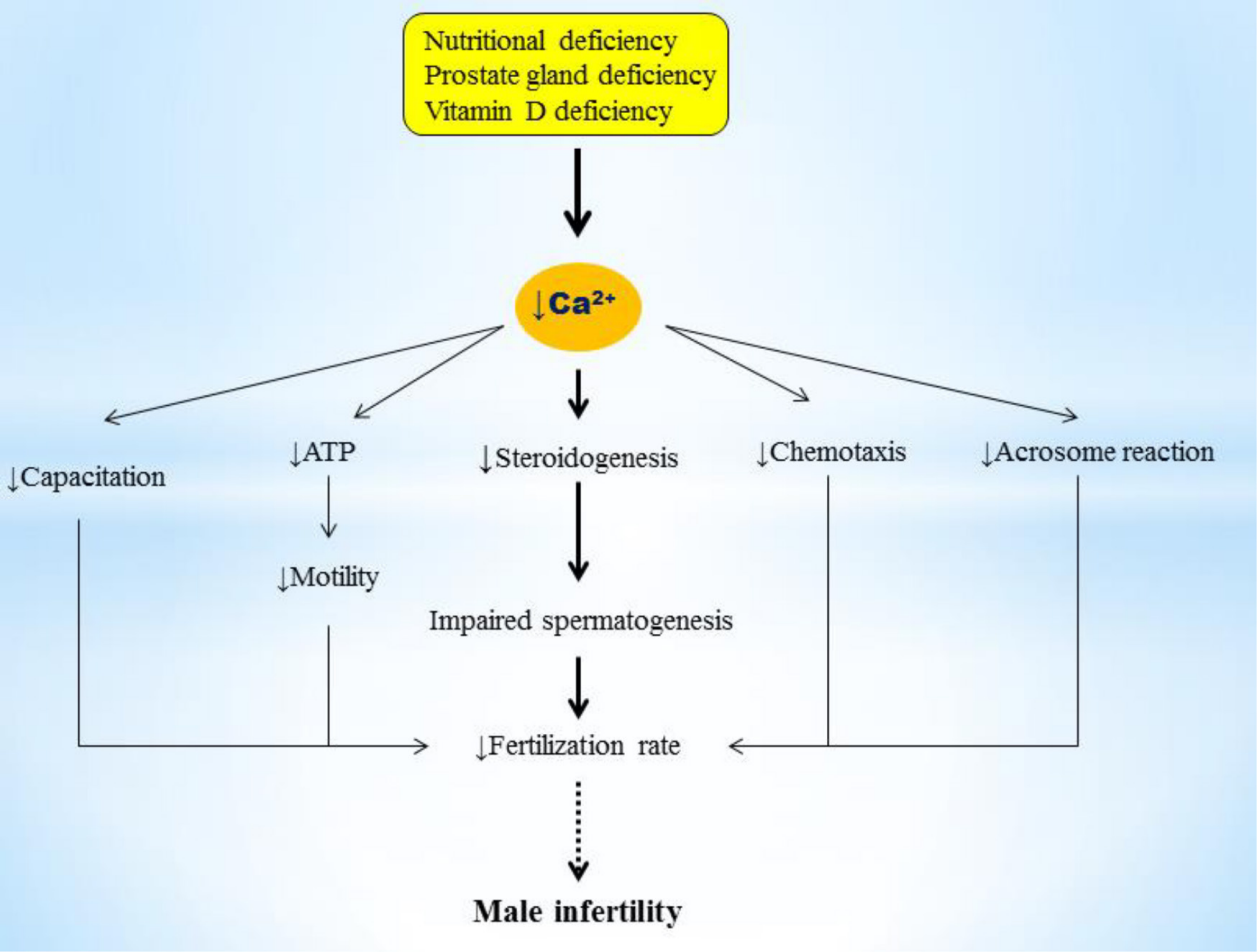
A schematic for the effects of Ca^2+^ deficiency on male infertility. Calcium deficiency decreases spermatogenesis and fertilization rate through several mechanisms including: reduced sperm chemotaxis, motility, capacitation, acrosome reaction, and steroidogenesis (21, 24, 39-42).

Although these studies showed relationship between reduced levels of Ca and increased risk of male infertility, the mechanism by which Ca deficiency affects fertilization rate of spermatozoa is not well-elucidated. The possible mechanisms in which Ca deficiency can affect sperm function and male infertility are discussed in the following sections. Impaired spermatogenesis, deficiency of steroidogenesis, poor sperm motility, abnormality in sperm chemotaxis, capacitation and acrosome reaction as well as reduced fertilization rate can be considered as possible mechanisms of the Ca deficiency effects on male infertility (Fig .1).

### Role of Ca in spermatogenesis

Spermatogenesis is a process in which diploid spermatogonial stem cells undergo meiosis and produce differentiated haploid spermatozoa (34). Several studies have pointed out that Ca has important role in the regulation of spermatogenesis and fertilization processes as well as growth, differentiation, proliferation and cell death in spermatogonium and spermatocyte cells (34, 35). Ultrastructural distribution of intracellular Ca has been illustrated within the various germ cells during different developmental stages of gametogenesis in rat testis (35). Interestingly, the Ca level increased significantly from early to late stages of spermatogenesis (spermatogonia>spermatocyte>spermatids>spermatozoa) and probably reflects changes in its homeostasis and specific function during the formation of spermatozoa (34-36). Similar results have been observed in rat seminiferous tubules that showed significant increase of Ca from spermatogonia to early spermatids and gradually at advanced stages of mouse spermatogenesis (37). Recent investigations have shown that Ca deposits are observed in the Sertoli, myoid, and Leydig cells (35). Interestingly, calmodulin is particularly abundant in the testis, suggesting the importance of Ca for normal spermatogenesis (38).

There are many Ca channels in germ cells of spermatogonia and spermatozoa which likely are responsible in the regulation of Ca signaling process (23). Ca signaling is necessary to shift from a proliferating phase of spermatogonia to the other advanced phases of spermatogenesis (43). Therefore, activation of the different Ca channels may induce changes in the Ca balance and homeostasis during different developmental stages of spermatogenesis (44). These changes are important in maintenance of spermatocyte function; otherwise any prolonged increase in the free Ca levels is toxic for cell function including chromatin condensation, mitochondria damage and activation of degradative enzymes so that ultimately can leads to cell death (45).

### Role of Ca in Leydig and Sertoli cells

Recent evidences have reported that Ca is essential for stimulation of steroidogenesis in Leydig cells of the testis (42). An increased Ca level has been shown to be associated with an increased production and secretion of testosterone in stimulated Leydig cell, while Ca chelators inhibit steroid synthesis (24). Ca also plays a critical role in Sertoli-Sertoli junction dynamics. Previous studies reported that testosterone-mediated enhancement of Ca is required for the influx of extracellular Ca in Sertoli cells, indicating that Ca channels in plasma membrane play crucial role in testosterone-Ca signaling (46). Therefore, given the regulatory function of testosterone in gonadal formation, differentiation, maturation and spermatogenesis, Ca deficiency can be associated with depletion of testosterone, inhibition of spermatogenesis and subsequently male infertility (Fig .1).

### Ca and sperm parameters

There are evidences that changes in intracellular and seminal plasma Ca can affect sperm function and motility. Spermatozoa modulate their movement in response to an alteration in the intracellular Ca concentration, depending on the pH of the medium (47). Numerous studies demonstrated a relationship between Ca and sperm motility (48). Given the biochemical requirements for Ca by adenosine triphosphate (ATP) to drive the flagella, the relationship between Ca and sperm motility seems to be logical (Fig .1). For example, a pervious study showed that addition of Ca with calsemin to isolated ram caudal spermatozoa caused stimulation of flagellar beat activity (49). Morton et al. (50) demonstrated that reduced seminal Ca concentrations are positively correlated with decreased human sperm motility in the epididymal. Similarly, Schmid et al. (14) reported that declined level of intracellular Ca in sperm is significantly associated with increased risks of poor sperm motility, independent of male age.

Banjoko and Adeseolu (51) observed that men with hypomotile sperm (<60%) exhibited lower Ca concentrations compared to men with normal motility. Furthermore, seminal plasma Ca was negatively correlated with sperm motility and count. Bassey et al. (48) observed that seminal plasma Ca level was significantly lower in oligospermic, azoospermic and asthenoligospermic infertile men compared to normospermic men. A more recent study has found a positive correlation between seminal plasma Ca concentration and semen parameters including pH, volume, sperm counts, and HOST% (52). Experimental evidences showed that use of EDTA, as a Ca chelator, causes a reduction in Ca concentration and significant loss of sperm motility (53). This suggests the regulatory effect of Ca on sperm motility and destructive effect of EDTA on spermatozoa. In another experimental study, Uhland et al. (54) showed that 1,25(OH)2D3 treatment increased intracellular Ca concentration in human spermatozoa from an intracellular Ca storage, increased sperm motility and induced the acrosome reaction.

### Sperm chemotaxis

Sperm chemotaxis is a process in which spermatozoa are attracted toward egg (55). This is an important event in the fertilization process and may provide an insight into the mechanism underlying sperm-egg interaction. Recent studies have indicated that Ca may play a central role in sperm chemotaxis because it regulates sperm flagellar beating (56). When the spermatozoon ejaculated into the female reproductive tract, progesterone stimulates Ca entry from extracellular spaces into the spermatozoon. The increased intracellular Ca induces the beating of sperm flagella, and subsequently causes to the chemotactic turn and ‘turn-and-straight’ movements (35).

### Role of Ca in sperm capacitation

Capacitation is a process in which sperm cells undergo a series of consecutive biochemical and molecular events during their movement across the female reproductive tract before reaching and fusing with oocyte. Sperm capacitation is modulated by several molecules present in the female reproductive tract. Ca is now considered as one of the significant factors that regulate sperm capacitation. Increased level of intracellular Ca as well as membrane hyperpolarization has been reported during the spermatozoon capacitation (36). Recent studies have indicated that Ca modulates sperm capacitation via the regulation of sperm cAMP-dependent signaling and tyrosine phosphorylation pathways in a biphasic manner (34). It has been also shown that the distribution of intracellular Ca is remodeled during post-mating capacitation in the male gamete of animals (57). In addition, recent proteomics studies identified some proteins such as ryanodine receptor, troponin, and sarcoplasmic Ca-binding protein that may contribute to the processes of Ca signaling in the male gamete of animals during different stages of reproduction such as capacitation (58, 59).

### Sperm acrosome reaction

The acrosome reaction is a critical step during sperm interaction. This is a process in which spermatozoa penetrate and fuse with the oocyte membrane. Many studies indicated that Ca influxe through the Ca channels of the sperm plasma membrane is necessary to initiate the acrosomal reaction and sperm fertility (37). This process is associated with the release of enzymes and membrane modifications, which are required for sperm-egg communication (60).

In a study, Walensky and Snyder (61) showed that acrosome is a source of internal Ca storage. Additionally, it has been demonstrated that both the plasma and the acrosomal membranes of mammalian spermatozoa contain Ca pumps which serve as a Ca storage during acrosome reaction (62). Experimental studies have shown that addition of ionophore A23187, which actively transports Ca from extracellular to intracellular space, induces sperm acrosome reaction (63). Another experimental study showed that the initiation of acrosome reaction with the ionophore is possible in the presence of Ca in the extracellular space (64). De Jonge (65) reported that exposure of capacitated spermatozoa to progesterone caused Ca influx from extracellular to intracellular space and initiation of sperm acrosome reaction. In another research, Benoff et al. (66) indicated that Cadmium (Cd) reduces sperm ability to undergo acrosome reaction via inhibition of Ca channels. Arabi and Mohammadpour (67) found that Cd can change the integrity and fluidity of acrosomal membranes of spermatozoa and induce abnormal acrosome reaction.

### Role of Ca in fertilization

A great number of studies demonstrated the critical role of Ca in the regulation of egg activation and successful fertilization (68). Upon fertilization an increase in intracellular Ca induces sperm movement across the egg in a global wave as a sign of egg activation. During the spermatozoon-egg interaction, Ca is injected into the egg that triggers signal transduction pathway and initiates egg activation (39). A more recent study has shown that a large amount of Ca is concentrated in the head of mature spermatozoa that facilitates Ca injection into the egg (38). The Ca wave triggered by the spermatozoon can be generated even when the egg or oocyte is placed in Ca-free media, suggesting that it is because of release from internal sources (69). Some studies demonstrated that changes in intracellular Ca levels are involved in both oocyte maturation and egg activation at fertilization process (70). Ca signalling also plays a key role in the development of patterning in early embryos (69).

## Conclusion

Ca deficiency can cause male infertility via several cellular and molecular mechanisms. Given the regulatory function of Ca in steroidogenesis, sperm motility, chemotaxis, capacitation and acrosome reaction within the women reproductive tract, Ca deficiency can be asEffects sociated with reduced fertilization rate and male infertility. These data indicate that infertile patients may benefit from Ca supplementation. Therefore, Ca level should be monitored in semen of men with idiopathic infertility.
